# Analysis of infiltrated immune cells in left atriums from patients with atrial fibrillation and identification of circRNA biomarkers for postoperative atrial fibrillation

**DOI:** 10.3389/fgene.2022.1003366

**Published:** 2022-12-09

**Authors:** Yubin Chen, Tianyu Ouyang, Yue Yin, Cheng Fang, Can-E Tang, Jingmin Luo, Fanyan Luo

**Affiliations:** ^1^ Department of Cardiac Surgery, Xiangya Hospital, Central South University, Changsha, China; ^2^ Department of Endocrinology, Xiangya Hospital, Central South University, Changsha, China; ^3^ The Institute of Medical Science Research, Xiangya Hospital, Central South University, Changsha, China; ^4^ Department of Cardiology, Xiangya Hospital, Central South University, Changsha, China; ^5^ National Clinical Research Center for Geriatric Disorders, Xiangya Hospital, Central South University, Changsha, China

**Keywords:** atrial fibrillation, postoperative atrial fibrillation, immune cells, inflammation, circRNA, biomarker

## Abstract

**Background:** Atrial fibrillation (AF) increases the risk of stroke and heart failure. Postoperative AF (POAF) increases the risk of mortality after cardiac surgery. This study aims to explore mechanisms underlying AF, analyze infiltration of immune cells in left atrium (LA) from patients with AF, and identify potential circular RNA (circRNA) biomarkers for POAF.

**Methods:** Raw data of GSE797689, GSE115574, and GSE97455 were downloaded and processed. AF-related gene co-expression network was constructed using weighted gene correlation network analysis and enrichment analysis of genes in relevant module was conducted. Gene set enrichment analysis (GSEA) and gene set variation analysis (GSVA) were applied to investigate pathways significantly enriched in AF group. Infiltration of immune cells was analyzed using single-sample GSEA. Differentially expressed genes (DEGs) between patients with or without AF were identified and competing endogenous RNA (ceRNA) networks of DEGs were constructed. To screen biomarkers for POAF, differentially expressed circRNAs (DEcircRNAs) between patients with or without POAF were identified. Intersection between DEcircRNAs and circRNAs in ceRNA networks of DEGs were extracted and circRNAs in the intersection were further screened using support vector machine, random forest, and neural network to identify biomarkers for POAF.

**Results:** Three modules were found to be relevant with AF and enrichment analysis indicated that genes in these modules were enriched in synthesis of extracellular matrix and inflammatory response. The results of GSEA and GSVA suggested that inflammatory response-related pathways were significantly enriched in AF group. Immune cells like macrophages, mast cells, and neutrophils were significantly infiltrated in LA tissues from patients with AF. The expression levels of immune genes such as CHGB, HLA-DRA, LYZ, IGKV1-17 and TYROBP were significantly upregulated in patients with AF, which were correlated with infiltration of immune cells. ceRNA networks of DEGs were constructed and has_circ_0006314 and hsa_circ_0055387 were found to have potential predictive values for POAF.

**Conclusion:** Synthesis of extracellular matrix and inflammatory response were main processes involved in development and progression of AF. Infiltration of immune cells was significantly different between patients with or without AF. Has_circ_0006314 and hsa_circ_0055387 were found to have potential predictive values for POAF.

## Introduction

Atrial fibrillation (AF) is one of the most common cardiac arrhythmia diseases, which affects 2%–3% of the Western population (Heijman et al., 2018). Traditional risk factors of AF include older age, coronary artery disease, male sex, hypertension, obesity, diabetes mellitus, and obstructive sleep apnea (Staerk et al., 2017). And there are studies revealed that AF exhibited a genetic predisposition (Palatinus and Das, 2015; Tucker et al., 2016). AF could increase the risk of stroke, heart failure, and dementia (Michaud and Stevenson, 2021). These severer complications of AF significantly increased the mortality by a factor of 2.4 among men and by a factor of 3.5 among women (Benjamin et al., 1998).

Structural remodeling and electrical remodeling are the key pathogenesis of AF. Development of atrial fibrosis and enlargement of left atrium are the hallmark of structural remodeling and atrial fibrosis is considered to be the substrate for AF initiation and maintenance (Dzeshka et al., 2015). Atrial fibrosis is characterized by progressive accumulation of extracellular matrix (ECM) in myocardium, which is predominantly comprised of collagen (Ueha et al., 2012). Cardiac fibroblasts (CFs) account for up to 60% of cells in cardiac muscle and play pivotal roles in synthesis and formation of ECM (Souders et al., 2009). When activated by stimulators of collagen synthesis like transforming growth factor beta-1 (TGF-β1), CFs differentiate into myofibroblasts which have approximate 2-fold higher capacity to synthesize collagen than CFs (Baum and Duffy, 2011). The differentiation of CFs and the synthesis of collagen eventually lead to atrial fibrosis. Electrical remodeling of AF is associated with the dysregulation of cardiac ion channels such as Ca2+ and K+ channels (Christ et al., 2004). The altered expression or distribution of connexin-40 and connexin-43 could result in heterogeneous conduction and hampering cell to cell communication (Yan et al., 2018). In addition, inflammation is closely related to AF initiation and maintenance. Macrophages and T cells were found infiltrating in the atrium of patients with AF (Yamashita et al., 2010). Researches indicated that the increasements of inflammatory cytokines like C-reactive protein (CRP), interleukin (IL)-1β, IL-6, and tumor necrosis factor (TNF)-α were correlated with the progression of AF (Li and Brundel, 2020). Furthermore, inflammation-related transcript factors like nuclear factor kappa B (NF-κB) could upregulate the expression level of TGF-β1 and slow conduction by decreasing the expression of sodium channel, both of which accelerate the progression of AF (Ziolo and Mohler, 2015; Faria and Persaud, 2017). But the exact mechanism underlying AF initiation and maintenance is largely unknown and needs further exploration.

Postoperative AF (POAF) is characterized by new onset AF in the immediate period after cardiac or non-cardiac surgery, which increases the intensive care unit time and in hospital cost (Greenberg et al., 2017). In this study, we focused on POAF after cardiac surgery. POAF is an important complication of coronary artery bypass grafting and valvular surgery. And the prevalence of POAF ranges between 20% and 40% in different studies (Dobrev et al., 2019). According to recent researches, older age, male sex, congestive heart failure, hypertension, obesity, and cross-clamp time are independently associated with POAF (Dobrev et al., 2019). POAF significantly increases the risk of stroke, subsequent AF recurrence, and cardiovascular death (Ahlsson et al., 2010). Animal experiments suggested that dysregulation of autonomic nervous system, inflammation, and oxidative stress were related with POAF (Dobrev et al., 2019). However, the mechanism of POAF remains unclear. Therefore, it is hard to predict and prevent POAF.

This study aimed to construct AF-related gene co-expression networks, analyze the infiltration of immune cells in AF, construct competing endogenous RNA (ceRNA) networks of differentially expressed genes (DEGs), and identify potential biomarkers for prediction of POAF using bioinformatics.

## Materials and methods

### Data acquisition and processing

**FIGURE 1:**
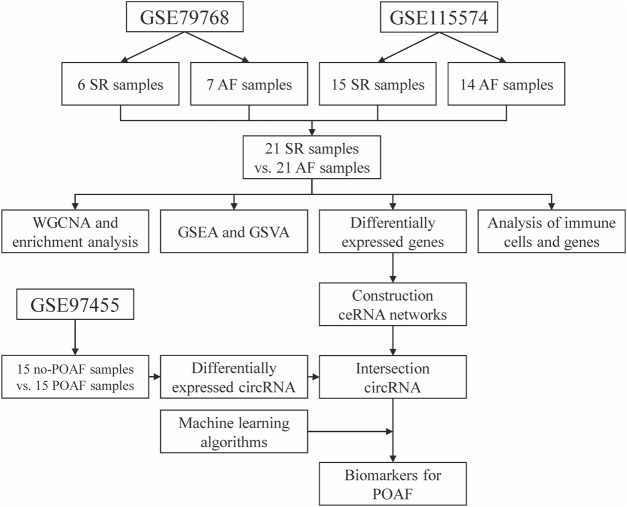
Flow chart diagram for the analysis process in this study. SR: sinus rhythm, AF: atrial fibrillation, POAF: postoperative atrial fibrillation, WGCNA: weighted gene correlation network analysis, GSEA: gene set enrichment analysis, GSVA: gene set variation enrichment analysis, ceRNA: competing endogenous RNA.

The analysis process of this study is shown in Figure 1. The raw data of GSE79768 (including six left atrium samples from patients with sinus rhythm (SR) and seven left atrium samples from patients with AF) and GSE115574 (including 15 left atrium samples from patients with SR and 14 left atrium samples from patients with AF) were downloaded from the Gene Expression Omnibus (GEO) database (https://www.ncbi.nlm.nih.gov/gds). These datasets were based on the same platform, GPL570 (Affymetrix Human Genome U133 Plus 2.0 Array). The expression matrix was extracted using “affy” package in R software (version 4.1.2; R Foundation for Statistical Computing, Vienna, Austria) and the data were annotated using the “dplyr” and “limma” packages in R software (version 4.1.2). After annotation, the data in expression matrix were normalized using “limma” package in R software (version 4.1.2). Then, the expression matrix of two datasets was merged and the batch effect between two datasets was removed using the “sva” package in R software (version 4.1.2). The merged expression matrix was used for further analysis.

The raw data of GSE97455 (including 15 plasma samples from patients without POAF and 15 plasma samples from patients with POAF) were downloaded from GEO database (https://www.ncbi.nlm.nih.gov/gds). In GSE97455, plasma samples were collected from patients who were going to undergo coronary artery bypass grafting surgery. The time of collection was before the start of the surgery and the occurrence of AF was recorded after surgery. The data were annotated using the “dplyr” and “limma” packages in R software (version 4.1.2) and then normalized using “limma” package in R software (version 4.1.2). Finally, the names of circular RNA (circRNA) in the dataset were transformed into the format of circRNA ID in circBase (http://www.circbase.org/) for further analysis.

### Gene co-expression network construction by weighted gene correlation network analysis (WGCNA)

Gene co-expression networks were constructed using the WGCNA package in R software (version 4.1.2) (Langfelder and Horvath, 2008). Soft-thresholding power was used to construct a weighted adjacency matrix. Relationships between a single gene and others in the analysis were incorporated, and the adjacency matrix was transformed into the topological matrix (TOM). Then, a hierarchical clustering analysis of genes was performed using 1—TOM as the distance measure. Thereafter, modules were detected using a dynamic tree cut algorithm with a minimum module size of 50 and a minimum cut height of 0.99. The correlation between each module and AF was calculated and shown in a heatmap. Finally, the most relevant gene modules were selected for further analysis.

### Gene ontology (GO) and kyoto encyclopedia of genes and genomes (KEGG) pathway enrichment analysis of genes in the relevant gene modules

The Database for Annotation, Visualization and Integrated Discovery (DAVID, 2021 Update, https://david.ncifcrf.gov/home.jsp) were used to conduct GO and KEGG pathways enrichment analysis of genes in the relevant gene modules.

### Gene set enrichment analysis (GSEA) and gene set variation analysis (GSVA)

The gene set files used in this study were downloaded from the Molecular Signatures Database version 7.5.1 (http://www.gsea-msigdb.org/gsea/msigdb/index.jsp). The enrichment scores of GO and KEGG pathways terms in each group were calculated using the GSEA software (version 4.2.3), and terms enriched in the AF group were identified. A nominal *p* value of <0.05 and false-discovery rate q value of <0.25 were considered as significantly enriched in the AF group.

GSVA was applied to evaluate GO and KEGG pathway terms enriched in each sample by converting the gene expression matrix into a gene set expression matrix using the “GSVA” package in R (version 4.1.2). After that, the differentially enriched terms between two groups were identified using R (version 4.1.2) with the threshold of *p* < 0.05. The differentially enriched terms were visualized using the “pheatmap” package in R (version 4.1.2).

### Single-sample gene set enrichment analysis (ssGSEA)

The gene set files used in this study were downloaded from the Molecular Signatures Database version 7.5.1 (http://www.gsea-msigdb.org/gsea/msigdb/index.jsp). The scores of 16 immune cells and four cardiac cells were calculated using “gsva” package in R (version 4.1.2).

### Identification of differentially expressed genes (DEGs)

DEGs and differentially expressed circRNA (DEcircRNA) between two groups were identified using R (version 4.1.2) with the threshold of *p* < 0.05 and absolute value of log fold change (FC) > 0.5. The immune and inflammation-related genes (IRGs) list was downloaded from Immport (https://www.immport.org/home). And differentially expressed IRGs (DEIRGs) were identified using R (version 4.1.2) with the threshold of *p* < 0.05 and absolute value of log fold change (FC) > 0.2.

### Construction of competing endogenous RNA (ceRNA) networks of DEGs

The Encyclopedia of RNA Interactomes (ENCORI, https://starbase.sysu.edu.cn/) (Li et al., 2014) database was used to construct ceRNA networks of DEGs. The micro-RNA (miRNA) which could interact with the DEGs was predicted using ENCORI with the threshold of number of supporting crosslinking-immunoprecipitation and high-throughput sequencing (CLIP-seq) experiments≥2 and number of target-predicting programs≥2. Then, the interaction pairs of miRNA-circRNA were predicted using ENCORI with the threshold of number of supporting CLIP-seq experiments≥5 and supporting degradome-seq experiments≥1. Finally, the circRNA-miRNA-mRNA interaction network was visualized using Cytoscape software (version 3.9.1).

### Identification of candidate circRNA biomarkers for POAF

The intersection between circRNA in ceRNA network and DEcircRNA was extracted. Upregulated circRNA which is associated with upregulated DEGs was selected as candidate biomarker for POAF. Similarly, downregulated circRNA which is associated with downregulated DEGs was selected as candidate biomarker for POAF.

### Construction of predictive model for POAF and identification of biomarkers for POAF

The candidate circRNAs were selected as features to construct predictive models. Support vector machine (SVM), random forest (RF), and neural network were applied to construct predictive models for POAF using “e1071”, “randomForest”, and “neuralnet” packages in R (version 4.1.2). Feature importance of each predictive model was analyzed and ranked. Thereafter, the intersection of top five circRNA in each predictive model was identified as potential biomarkers for POAF.

### Statistical analysis

The relative expression levels of mRNA were presented as mean ± standard deviation (SD). The difference in scores of immune cells and cardiac cells between different groups was analyzed by the Mann-Whitney *U* test. Pearson’s correlation analysis was used to analyze the relationship between inflammation genes and the scores of immune cells and cardiac cells. A value of *p* < 0.05 was considered to be statistically significant. Statistical analyses were performed using SPSS version 19 (IBM Corporation, Armonk, NY, United States) and R (version 4.1.2).

## Results

### Construction of AF-related gene co-expression networks and enrichment analysis of genes in the relevant modules

**FIGURE 2:**
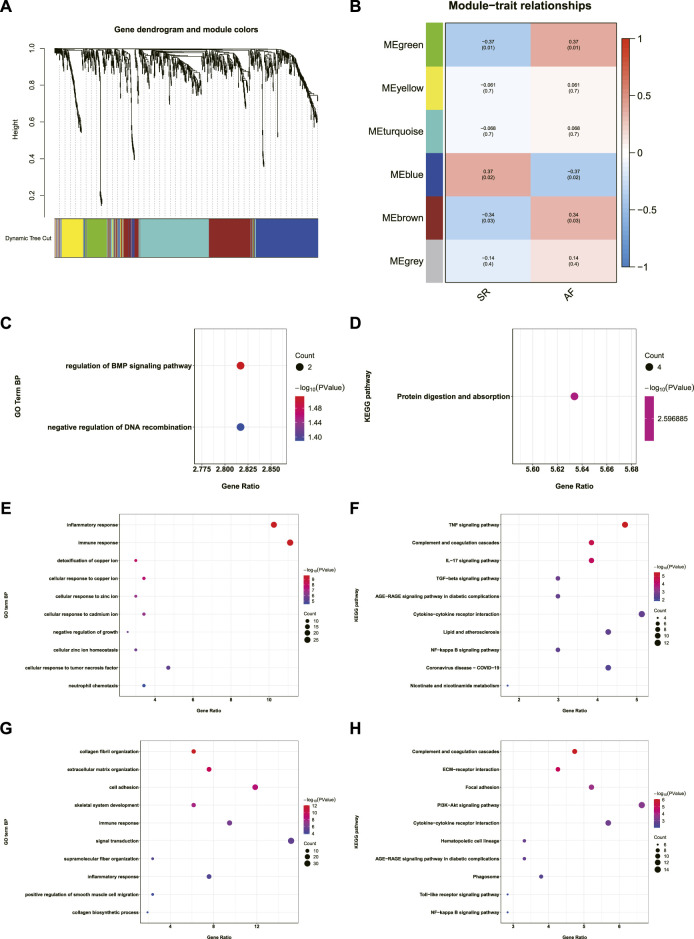
Construction of AF-related gene co-expression network and the enrichment analysis of genes in the AF-related gene modules. **(A)** Dendrogram and clustering for identification of gene co-expression modules. **(B)** Correlation analysis of gene co-expression modules with AF. The numbers above brackets were correlation coefficients and the numbers in brackets were *p* values. **(C,D)** GO-BP and KEGG enrichment analysis of genes in green module. **(E,F)** GO-BP and KEGG enrichment analysis of genes in blue module. **(G,H)** GO-BP and KEGG enrichment analysis of genes in brown module. AF: atrial fibrillation, GO-BP: gene ontology biological process, KEGG: Kyoto Encyclopedia of Genes and Genomes.

AF-related gene co-expression networks were constructed using WGCNA and six gene modules were obtained (Figure 2A). The correlation between each gene module and the occurrence of AF was analyzed, and coefficients and *p* value are shown in Figure 2B. The absolute values of coefficients of green, blue, and brown modules were close, therefore, enrichment analysis of genes in these modules were conducted, respectively. Genes in green module were significantly enriched in regulation of bone morphogenetic protein (BMP) signaling pathway, negative regulation of DNA recombination, and protein digestion and absorption (Figures 2C,D). The enrichment analysis of genes in blue module revealed that the top three terms in the GO biological process (BP) subdivision were inflammatory response, immune response, and detoxification of copper ion, while the top three KEGG pathways were tumor necrosis factor (TNF) signaling pathway, complement and coagulation cascades, and IL-17 signaling pathway (Figures 2E,F). The enrichment analysis of genes in brown module indicated that the top three terms in GO BP subdivision were collagen fibril organization, extracellular matrix organization, and cell adhesion, while the top three KEGG pathways were complement and coagulation cascades, ECM-receptor interaction, and focal adhesion (Figures 2G,H).

### GSEA and GSVA

**FIGURE 3:**
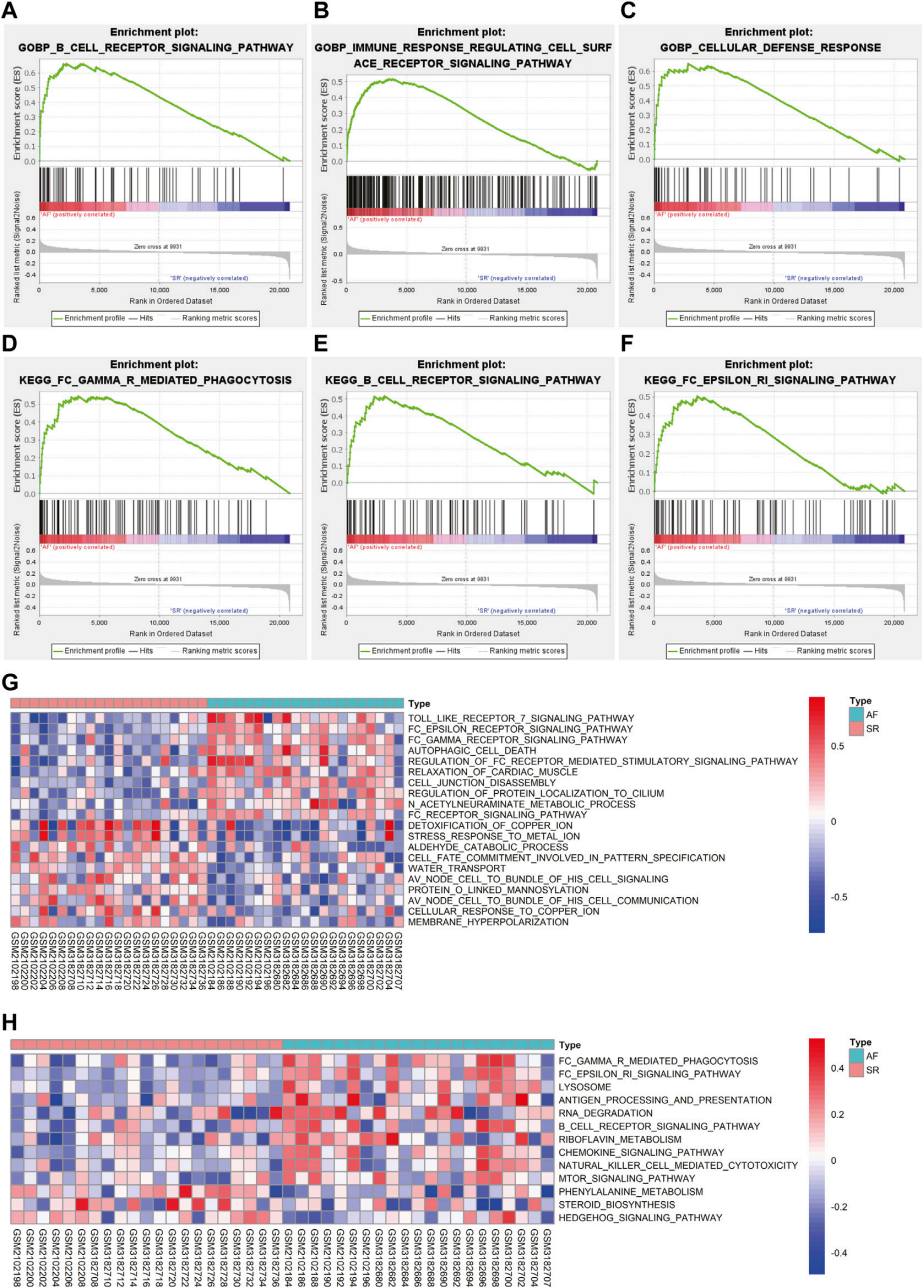
The results of GSEA and GSVA. **(A–C)** The results of GSEA revealed the GO-BP terms significantly enriched in AF group. **(D–F)** The results of GSEA revealed the KEGG pathways significantly enriched in AF group. **(G)** The variations of GO-BP terms between different samples were analyzed using GSVA and the top 20 significantly different GO-BP terms were shown in the heatmap. **(H)** The variations of KEGG pathways between different samples were analyzed using GSVA and the significantly different KEGG pathways were shown in the heatmap. GSEA: gene set enrichment analysis, GSVA: gene set variation analysis, GO-BP: gene ontology biological process, KEGG: Kyoto Encyclopedia of Genes and Genomes, AF: atrial fibrillation.

GSEA was conducted to further explore the terms of GO BP and pathways enriched in AF group. As shown in Figures 3A–C, the terms of GO BO like B cell receptor signaling pathway, immune response regulating cell surface receptor signaling pathway, and cellular defense response were significantly enriched in AF group. In terms of KEGG pathway, FC gamma R mediated phagocytosis, B cell receptor signaling pathway, and FC epsilon RI signaling pathway were significantly enriched in AF group (Figures 3D–F).

GSVA was utilized to analyze the variations of pathway between different samples and the differentially activated pathways were identified. The top 10 upregulated and downregulated terms of GO BP were displayed in Figure 3G. The results indicated that terms like Toll like receptor seven signaling pathway and FC epsilon receptor signaling pathway were significantly activated in AF group while detoxification of copper ion and stress response to metal ion were significantly downregulated in AF group (Figure 3G). The heatmap of variations of KEGG pathway between different samples was shown in Figure 3H suggesting the activation of FC gamma R mediated phagocytosis and FC epsilon RI signaling pathway in samples with AF, which is consistent with the results of GSEA.

### The infiltration of immune cells in different samples and the correlation between immune-related genes and immune cells

**FIGURE 4:**
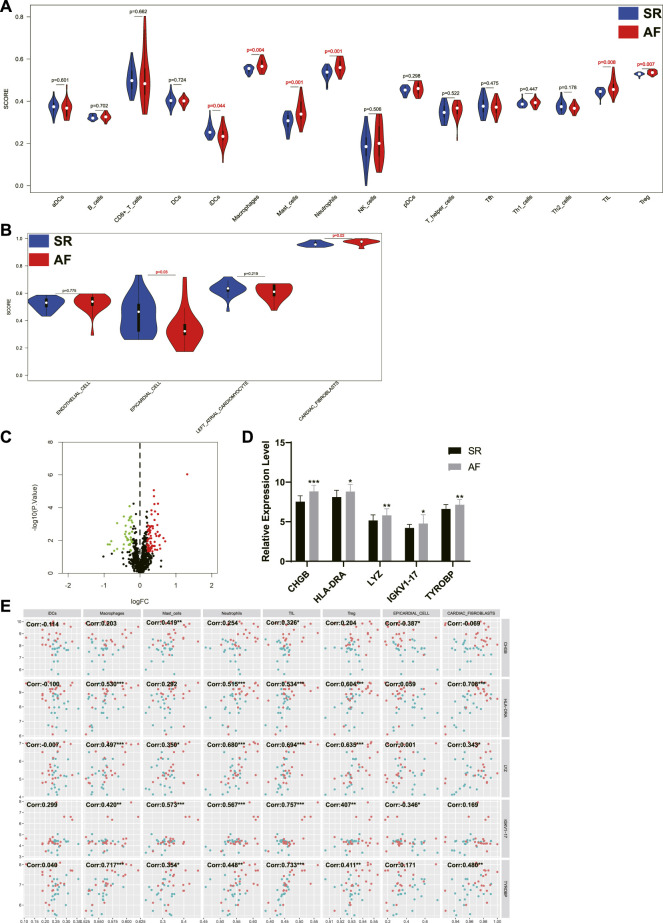
The analysis of immune cells and cardiac cells in LA samples and the correlation between immune genes, immune cells, and cardiac cells. **(A)** The scores of immune cells in LA samples. **(B)** The scores of cardiac cells in LA samples. **(C)** The volcano plot of differentially expressed immune genes between patients with or without AF. Dots in green: downregulated gene, dots in red: upregulated gene. **(D)** The expression levels of top five upregulated immune genes in SR group and AF group. **(E)** The correlation between immune genes, immune cells, and cardiac cells. Dots in blue: SR samples, dots in red: AF samples. LA: left atrium, SR: sinus rhythm, AF: atrial fibrillation. *, *p* < 0.05, **, *p* < 0.01, ***, *p* < 0.001.

The infiltration of immune cells in different samples was investigated using ssGSEA and the results showed that the scores of macrophages, mast cells, neutrophils, tumor infiltrating lymphocyte (TIL), and regulatory T cells (Treg) were significantly higher in AF group, while the score of immature dendritic cells (iDCs) was significantly decreased in AF group (Figure 4A). In addition, scores of four cardiac cells were calculated using ssGSEA, and the results revealed that the score of epicardial cell was significantly decreased in AF group, while the score of cardiac fibroblasts was significantly increased in AF group. Then, the differentially expressed immune-related genes (DEIRGs) between SR group and AF group were identified (Figure 4C) and the top five upregulated DEIRGs were *CHGB*, *HLA-DRA*, *LYZ*, *IGKV1-17*, and *TYROBP* (Figure 4D). The correlation between DEIRGs and different types of cells was determined and shown in Figure 4E.

### Construction of ceRNA networks of DEGs

**FIGURE 5:**
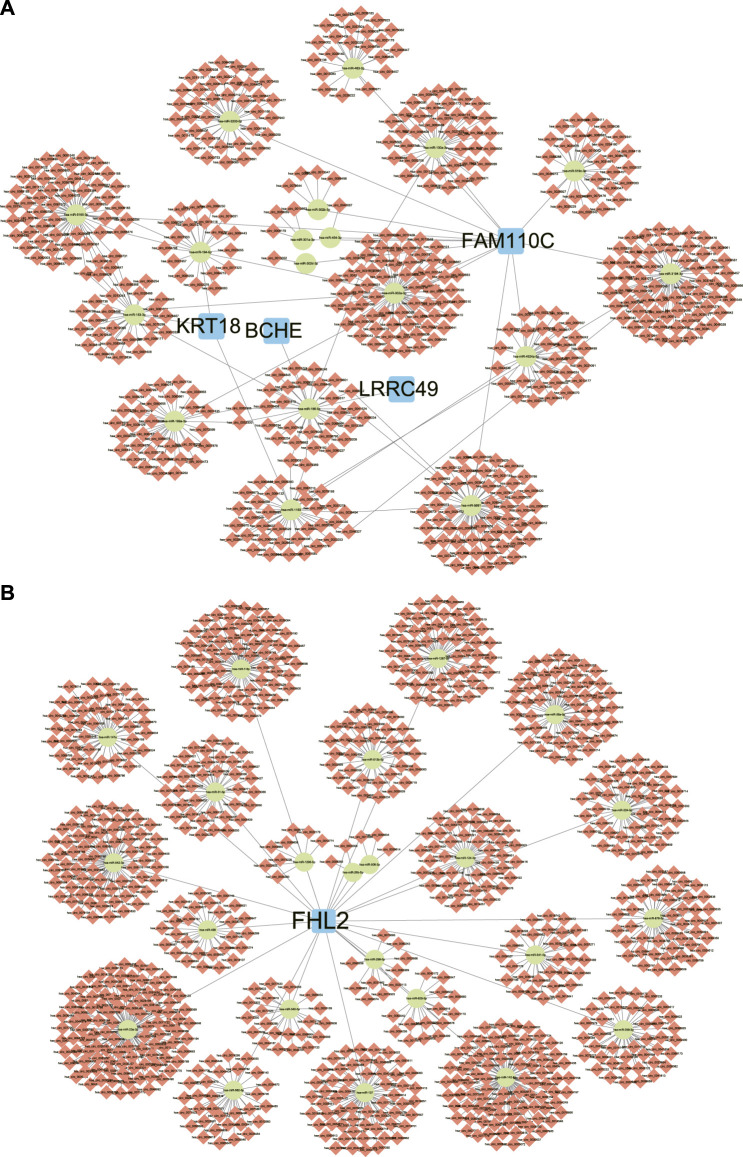
ceRNA networks of DEGs. **(A)** The ceRNA networks of downregulated DEGs. **(B)** The ceRNA networks of upregulated DEGs, FHL2. ceRNA: competing endogenous RNA, DEGs: differentially expressed genes.

The DEGs between SR group and AF group were analyzed and the top 10 upregulated and downregulated genes were shown in Table 1. Out of top 10 downregulated DEGs, ceRNA networks of *BCHE*, *FAM110C*, *KRT18*, and *LRRC49* were constructed successfully (Figure 5A). The prediction results of other six genes showed that there was no interaction pair of miRNA-mRNA according to the threshold of prediction. Similarly, out of top 10 upregulated DEGs, ceRNA networks of *FHL2*, *RELN*, and *LBH* were constructed successfully and the ceRNA network of *FHL2* was displayed in Figure 5B as representative network. miRNA which could interact with other seven upregulated genes was not found with the threshold of prediction.

**TABLE 1:** The expression levels of top 20 DEGs between SR and AF group.

Gene	SR group	AF group	Log FC	*p* Value
CHGB	7.54 ± 0.75	8.84 ± 0.77	1.32	<0.001
TRDN-AS1	4.16 ± 0.59	5.43 ± 0.88	1.27	<0.001
ATP1B4	3.87 ± 0.79	4.92 ± 1.06	1.05	<0.001
FHL2	8.85 ± 0.88	9.86 ± 1.12	1.02	0.001
RELN	7.16 ± 1.12	8.18 ± 1.04	1.02	0.003
DHRS9	8.19 ± 0.84	9.15 ± 0.59	0.98	<0.001
LBH	6.86 ± 0.65	7.82 ± 0.61	0.98	<0.001
MYOZ1	6.59 ± 1.28	7.49 ± 1.30	0.91	0.022
COMP	5.18 ± 0.84	6.08 ± 1.54	0.91	0.018
IGFBP2	9.18 ± 0.95	10.05 ± 0.92	0.89	0.002
MSLN	6.08 ± 1.53	4.72 ± 1.39	−1.33	0.003
PRG4	6.38 ± 1.87	5.04 ± 2.12	−1.22	0.034
C1orf105	7.39 ± 0.79	6.22 ± 0.69	−1.15	<0.001
BCHE	7.58 ± 0.72	6.46 ± 0.91	−1.12	<0.001
C19orf33	6.13 ± 1.27	5.05 ± 0.91	−1.08	0.002
AKAP3	6.80 ± 0.97	5.73 ± 0.93	−1.06	<0.001
FAM110C	6.64 ± 1.05	5.58 ± 0.71	−1.06	<0.001
HAS1	6.34 ± 1.25	5.29 ± 0.97	−1.05	0.003
LRRC49	8.12 ± 0.54	7.09 ± 0.74	−1.03	<0.001
KRT18	7.91 ± 1.08	6.89 ± 1.01	−1.02	0.002

Abbreviation: DEGs: differentially expressed genes, SR: sinus rhythm, AF: atrial fibrillation, FC: fold change.

### Screening of candidate circRNA biomarkers for POAF

**FIGURE 6:**
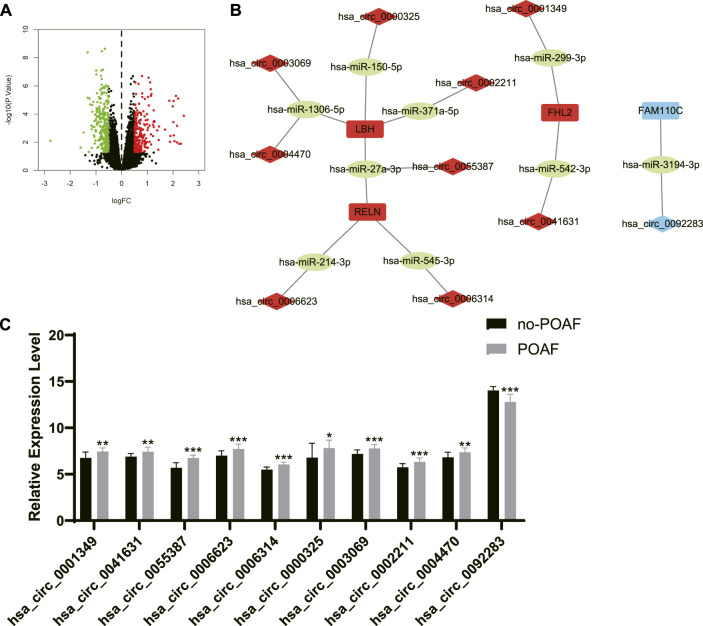
Screening of candidate circRNA biomarkers for POAF. **(A)** The volcano plot of differentially expressed circRNAs between patients with or without POAF. Dots in green: downregulated circRNAs, dots in red: upregulated circRNAs. **(B)** The ceRNA networks of DEGs after screening. The label in red: upregulated DEGs or differentially expressed circRNAs, the label in blue: downregulated DEGs or differentially expressed circRNAs, the label in green: miRNAs. **(C)** The expression levels of candidate circRNA biomarkers for POAF in patients with or without POAF. POAF: postoperative atrial fibrillation, DEGs: differentially expressed genes. *, *p* < 0.05, **, *p* < 0.01, ***, *p* < 0.001.

The differentially expressed circRNA (DEcircRNA) between no-POAF group and POAF group was identified with the threshold of *p* < 0.05 and absolute value of log fold change (FC) > 0.5 (Figure 6A). There were 210 upregulated circRNA and 293 downregulated circRNA. The intersection between upregulated circRNA and the circRNA in ceRNA networks of upregulated DEGs was extracted as candidate circRNA biomarkers for POAF, and the intersection between downregulated circRNA and the circRNA in ceRNA networks of downregulated DEGs was extracted as candidate circRNA biomarkers for POAF, too (Figure 6B). Finally, 10 candidate circRNA biomarkers for POAF were obtained and the expression levels of these circRNA in different groups of GSE97455 were shown in Figure 6C.

### Construction of predictive models for POAF and identification of biomarkers for POAF

**FIGURE 7:**
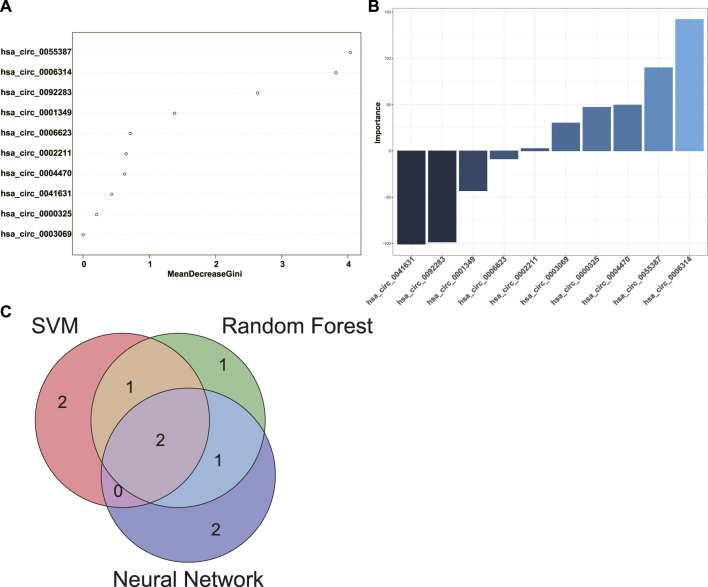
Identification of biomarkers for POAF. **(A)** The rank of feature importance in RF predictive model for POAF. **(B)** The rank of feature importance in neural network predictive model for POAF. **(C)** The intersection between top five features of each model was shown in the Venn diagram. POAF: postoperative atrial fibrillation, RF: random forest.

10 candidate circRNA biomarkers for POAF were selected as features of predictive models. SVM, RF, and neural network were applied to construct predictive models for POAF based on the data of GSE97455. In order to achieve highest accuracy with least features in model, the algorithm of SVM chose hsa_circ_0006314, hsa_circ_0001349, hsa_circ_0055387, hsa_circ_0003069, and hsa_circ_0002211 out of 10 candidate circRNA biomarkers as features of predictive model (accuracy = 1). The algorithm of RF used 10 candidate circRNA biomarkers as features of predictive model (accuracy = 0.933) and the rank of feature importance was shown in Figure 7A. Top five features in the RF model were hsa_circ_0055387, hsa_circ_0006314, hsa_circ_0092283, hsa_circ_0001349, and hsa_circ_0006623. Similarly, the algorithm of neural network selected 10 candidate circRNA biomarkers to construct predictive model (accuracy = 1). The importance of features in neural network model was analyzed and the top five features were hsa_circ_0006314, hsa_circ_0041631, hsa_circ_0092283, hsa_circ_0055387, and hsa_circ_0004470 (Figure 7B). Finally, the intersection between top five features of three models was extracted. Hsa_circ_0006314 and hsa_circ_0055387 were identified as potential biomarkers for POAF.

## Discussion

AF is the most common cardiac arrhythmia disease which is characterized by irregular and abnormally fast contraction of atrial cardiomyocytes (Hindricks et al., 2020). Symptoms of AF include irregular heart rate, palpitation, dizziness, and tiredness (Hindricks et al., 2020). Moreover, AF could lead to severer complications like stroke, heart failure, and cognitive impairment, both of which are associated with the increasement of morbidity and mortality (Zoni-Berisso et al., 2014). According to the epidemiological study, the prevalence of AF would keep increasing in the next 3 decades (Colilla et al., 2013). It was suggested that by the year 2050, approximately 5.2 million men and 3.1 million women with age older than 60 would suffer from AF in China (Zhang et al., 2021). AF is a serious threat to patients. Therefore, it is necessary to further explore the mechanisms underlying AF initiation and maintenance and find out potential therapeutic targets for AF.

As mentioned above, atrial structural remodeling is the key substrate of AF. Left atrium (LA) and right atrium (RA) exhibited different susceptibilities in AF initiation and maintenance (Tsai et al., 2016). Research revealed that the high frequency reentrant sources (or rotors) were more distributed in LA (Kalifa et al., 2006). Besides, Sahadevan et al. reported that the dominant frequency of AF was present in LA more frequently (Sahadevan et al., 2004). Moreover, Lin et al. demonstrated that complex fractionated atrial electrograms were more extensively present in LA (Lin et al., 2009). There were other researches suggested that LA is more important than RA in AF maintenance (Haissaguerre et al., 2008; Oral et al., 2008). In consideration of the importance of LA in AF initiation and maintenance, gene expression data of LA samples from patients with or without AF were extracted from GSE79768 and GSE115574 to investigate the potential mechanisms of AF. After construction of AF-related gene co-expression networks, the results of correlation analysis between each gene module and the presence of AF indicated that the green, blue, and brown modules were significantly relevant to AF. Genes in green module were significantly enriched in regulation of BMP signaling pathway. BMPs are members of TGF-β family and involve in the control of cell behavior, proliferation, and differentiation (Kraj, 2022). Chen et al. reported that BMP7 could inhibit atrial fibrosis *via* mediating TGF-β1/SMAD3 signaling pathway (Chen et al., 2016). In terms of blue and brown modules, enrichment analysis revealed that genes in these modules were significantly associated with synthesis and organization of ECM and inflammatory response. Accumulation of ECM in atrium is the key feature of atrial fibrosis which is mainly mediated by TGF-β1 signaling pathway, renin-angiotensin-aldosterone axis, matrix metalloproteinases (MMPs), and tissue inhibitors of MMPs (Sohns and Marrouche, 2020). CFs are the pivotal cell type in the synthesis and organization of ECM, which account for up to 60% of cells in the cardiac muscle (Souders et al., 2009). In this study, the score of CFs calculated by ssGSEA was significantly increased in AF group, further validating the important role of CFs in AF. In addition to affecting atrial structure, atrial fibrosis could lead to slow conduction, increase heterogeneity of conduction, and turn the atrium into the substrate of AF initiation (Li et al., 1999). Atrial fibrosis is also related to the occurrence and perpetuation of focal and re-entry arrhythmia, which contributes to the maintenance of AF (Burstein and Nattel, 2008). Thus, future research in therapy of AF should take atrial fibrosis into consideration to improve clinical outcome (Sohns and Marrouche, 2020).

There were researches demonstrated that patients with AF exhibited systemic inflammation with the increasement of inflammatory markers like CRP, IL-6, and IL-8 in peripheral blood (Hu et al., 2015). Inflammatory cytokines contribute to the development of electrical remodeling directly. TNF could induce dysregulated Ca^2+^ in pulmonary vein cardiomyocytes (Lee et al., 2007) and inhibit the expression level of sarcoplasmic/endoplasmic reticulum Ca^2+^ ATPase 2a (SERCA2a) (Kao et al., 2010). Moreover, it was reported that TNF could decrease the expression levels of connexin-40 and connexin-43 and alter the intracellular distribution of connexin-43 in mice model (Sawaya et al., 2007; Liew et al., 2013). Inflammation also involves in the development and progression of structural remodeling. Inflammatory cytokines like CRP, heat shock protein (HSP) 27, and IL6 are positively associated with the atrial size (Hu et al., 2015). TNF could mediate structural remodeling *via* activating TGF-β signaling pathway and upregulating the expression level of MMP2 and MMP9 (Liew et al., 2013). In this study, the infiltration of immune cells and DEIRGs were analyzed to further investigate the role of inflammatory response in AF. The results indicated that the scores of macrophages, mast cells, neutrophils, TIL, and Treg were significantly increased in AF group. S. Kugler et al. found that the infiltration of macrophage in LA was significantly increased in patients with AF (Kugler et al., 2022). Mast cell frequently infiltrates in the fibrotic tissue and is an important cell type in mediating tissue fibrosis (Legere et al., 2019). Inhibition of mast cell was able to rescue cardiac fibrosis of AF mouse model (Legere et al., 2019). Zhang et al. reported that the infiltration of neutrophils in atrium was associated with the increased vulnerability to AF in a canine sterile pericarditis model (Zhang et al., 2011). An increased ratio of neutrophil to lymphocyte in peripheral blood was related to the increased incidence of new onset AF in patients after surgery. But the roles of TIL and Treg in AF are unclear and need further investigation. After that, DEIRGs between SR group and AF group were identified and the top five upregulated DEIRGs were *CHGB*, *HLA-DRA*, *LYZ*, *IGKV1-17*, and *TYROBP*. The roles of these IRGs in AF need further exploration, and these IRGs might be potential therapeutic targets for AF.

MiRNAs are noncoding RNAs with length of 21–24 nucleotide and mediate gene expression by binding to the 3′-untranslated region of mRNA which inhibits mRNA translation or inducing mRNA degradation (Blazejowska et al., 2021). CircRNAs, new type of endogenous non-coding RNAs can form covalent closed-loop structures through reverse splicing with longer half-life than mRNA (Guo et al., 2022). Both of miRNAs and circRNAs involve in the development and progression of cardiac diseases and might be potential biomarkers for cardiac diseases (Lu et al., 2022). The theory of ceRNA network suggests that non-coding RNAs like circRNAs contain site for binding to miRNAs and circRNAs could sequester and inactive miRNAs by acting as miRNA sponge, which further mediates the expression level of target mRNA of miRNAs (Guo et al., 2022). The construction of ceRNA network would benefit to identification of potential therapeutic targets or biomarkers for diseases. In this study, ceRNA networks of DEGs were constructed. circRNAs in these networks might be associated with the formation of substrate for AF and have potential predictive values for AF, which need further validation.

POAF is a common complication after cardiac surgery which is associated with increased risk of subsequent AF and mortality after cardiac surgery (Ahlsson et al., 2010). POAF could also occur after non-cardiac surgery, but we only discussed POAF after cardiac surgery in this study. Anderson et al. reported that reactive oxygen species (ROS) were increased in the atriums from patients who were susceptible to POAF (Anderson et al., 2014). The expression level of NF-κB was significantly upregulated in atrial tissues from patients with POAF (Li et al., 2009). Both ROS and inflammatory factors are key mediators for development and progression of AF (Hu et al., 2015). Transient factors during cardiac surgery like activation of autonomic nervous system, inflammation, and cardiopulmonary bypass significantly contribute to the initiation of POAF (Maesen et al., 2012). Besides, pre-existing atrial substrate like atrial fibrosis, inflammation, and electrical remodeling in patients also involves in the presence of POAF (Dobrev et al., 2019). In summary, the occurrence of POAF is largely due to the acute transient factors during cardiac surgery and the pre-existing atrial substrates which facilitates the initiation of AF (Dobrev et al., 2019). Therefore, we speculated that circRNAs in the ceRNA networks of DEGs might have potential predictive values for POAF, but these circRNAs need to be further screened and validated. To screen circRNAs with potential predictive values for POAF, circRNAs expression data of GSE97455 which included 15 plasma samples from patients without POAF and 15 plasma samples from patients with POAF were used and DEcircRNAs between patients with or without AF were identified. Then, the intersection between DEcircRNAs and circRNAs in the ceRNA networks of DEGs was extracted. After that, Upregulated circRNAs which is associated with upregulated DEGs and downregulated circRNAs which is associated with downregulated DEGs were selected as candidate biomarkers for POAF and 10 circRNAs were obtained. In order to identify hub circRNAs out of 10 circRNAs, predictive models for POAF were constructed using 10 circRNAs as features by SVM, RF, and neural network, both of which are machine learning algorithms and had been used in diagnosis of cardiovascular disease (Ahsan and Siddique, 2022). Top five circRNAs of each predictive models were identified according to the feature importance ranked by algorithms. Finally, the intersection between top five circRNAs of each predictive model was extracted. Hsa_circ_0006314 and hsa_circ_0055387 were identified as hub circRNAs and might be potential biomarkers for POAF. According to the ceRNA networks, hsa_circ_0055387 might be able to affect the expression of LBH and RELN *via* binding has-miR-27a-3p, and hsa_circ_0006314 might mediate the expression of RELN by interacting with has-miR-545-3p. But the exact roles of hsa_circ_0006314 and hsa_circ_0055387 in POAF and predictive values of hsa_circ_0006314 and hsa_circ_0055387 for POAF need further validation.

In conclusion, synthesis of ECM and inflammatory response were main processes involved in the development and progression of AF. Immune cells like macrophages, mast cells, neutrophils, TIL, and Treg were significantly infiltrated in LA tissues from patients with AF. The expression levels of IRGs such as CHGB, HLA-DRA, LYZ, IGKV1-17, and TYROBP were significantly upregulated in patients with AF, which were also correlated with infiltration of immune cells in patients. After screening, hsa_circ_0006314 and hsa_circ_0055387 were found to have potential predictive values for POAF.

## Data Availability

Publicly available datasets were analyzed in this study. This data can be found here: GSE79768: https://www.ncbi.nlm.nih.gov/geo/query/acc.cgi?acc=GSE79768 GSE115574: https://www.ncbi.nlm.nih.gov/geo/query/acc.cgi?acc=GSE115574 GSE97455: https://www.ncbi.nlm.nih.gov/geo/query/acc.cgi?acc=GSE97455.
